# Successful coronary stenting in a patient with factor V deficiency in the absence of fresh frozen plasma transfusion: Case report

**DOI:** 10.1097/MD.0000000000009274

**Published:** 2017-12-15

**Authors:** Seongil Choi, MooKon Song

**Affiliations:** Department of Cardiology, Hanyang University Hanmaem Changwon Hospital, Changwon-si, Republic of Korea.

**Keywords:** blood transfusion, factor V deficiency, percutaneous coronary intervention, platelet aggregation inhibitor

## Abstract

**Rationale:**

Drug-eluting stent (DES) implantation in a patient with factor V deficiency (F5D) is very complex. No antithrombotic therapy study has been reported for F5D patients who undergo a coronary stenting procedure.

**Patient concerns:**

A 73-year-old woman presented with chest discomfort and exertional dyspnea. Coronary stenting was performed successfully using DES stents.

**Diagnoses:**

The D-dimer, prothrombin time, and partial thromboplastin time prolongation persisted from admission until 24 hours after coronary stenting. Epistaxis and blood-tinged sputum occurred on day 3. The antiplatelet therapy measured using a Multiplate Analyzer was adequate, and other laboratory findings except factor V activity (14%) were within normal ranges; she was diagnosed with F5D based on low factor V activity.

**Interventions:**

While taking 90 mg of ticagrelor and 100 mg of aspirin daily, the patient revisited due to recurrent epistaxis, hemoptysis, and coughing on day 26. Epistaxis and hemoptysis stopped after the aspirin was discontinued. Finally, the daily maintenance dose was reduced to 90 mg of ticagrelor once.

**Outcomes:**

She led healthy life for 9 months without any recurrent symptoms and the test results also were stabilized.

**Lessons:**

We report a case of an F5D patient who underwent coronary stenting in the absence of frozen fresh plasma transfusion who received successful maintenance therapy using a single antiplatelet agent (90 mg of ticagrelor/day) with recurrent multiple mucosal bleeding events after coronary stenting.

## 1 Introduction

Factor V deficiency (F5D) is a rare hematological disorder with an estimated incidence of 1 case per million people.^[1,2]^ Until now, more than 200 cases have been recorded worldwide in the literature.^[2]^ F5D patients present with various clinical manifestations. Although mucosal bleeding is the most common, fatal bleeding complications are also possible. Thus, F5D increases the difficulty of invasive testing, and surgical and procedural treatments. When long-term antithrombotic drugs, including antiplatelet agents and anticoagulants, are required in patients at high risk of bleeding, one of the biggest challenges is coronary intervention to treat coronary artery disease. Most studies recommend preinterventional or preoperative supplementation with fresh frozen plasma (FFP) to reduce bleeding risk.^[2,3]^ However, in addition to the bleeding risk caused by antithrombotic therapy, the hypercoagulable state in coronary intervention has an adverse effect on stent thrombosis, mortality, and prognosis during the postinterventional period.^[4]^ The contemporary standard therapy for significant coronary artery stenosis is implanting a drug-eluting stent (DES). However, because implanting a DES delays endothelial healing and requires long-term antithrombotic therapy, DES implantation in an F5D patient is very complex. No antithrombotic therapy study to date has been reported for F5D patients undergoing coronary stenting. Herein, we report a case of an F5D patient who underwent coronary stenting in the absence of an FFP transfusion and who received successful maintenance therapy using a single antiplatelet agent with recurrent multiple mucosal bleeding events after coronary stenting.

## 2 Case report

**Figure 1:**
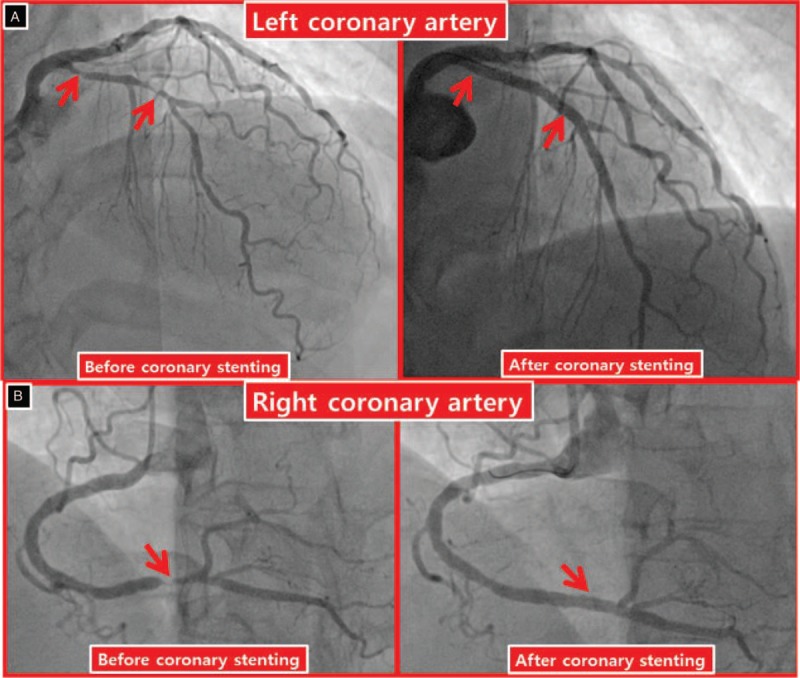
Coronary angiography. Significant stenoses are evident from the proximal to the mid-portion of the left anterior descending artery and the distal portion of the right coronary artery; the coronary artery vessel sizes were normalized after stenting.

**Figure 2:**
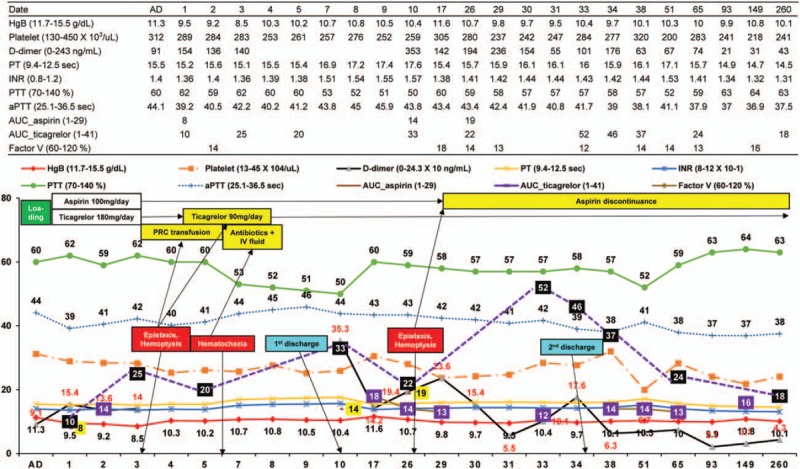
Clinical course and laboratory findings. The prolonged prothrombin time (PT) and partial thromboplastin time (PTT) values were consistent and factor V activity was consistently measured as low (15%); aspirin discontinuance abruptly increased the value of the area under the curve for ticagrelor from 22 to 52. AD = admission day, aPTT = activated PTT, AUC_aspirin = area under the curve for aspirin, AUC_ticagrelor = area under the curve for ticagrelor, HgB = hemoglobin, INR = international normalized ratio, IV = intravenous, PRC = packed red cell, PT = prothrombin time, PTT = partial thromboplastin time.

**Figure 3:**
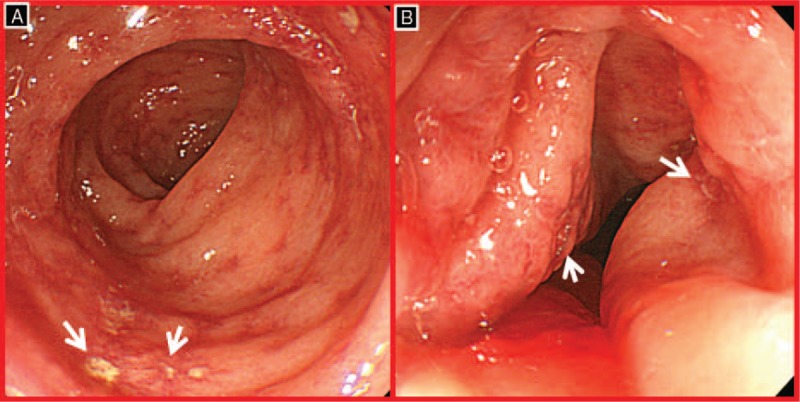
Sigmoidoscopic findings. Sigmoidoscopy showed mucosal friability, petechial hemorrhage, and discrete ulcers with surrounding edema.

A 73-year-old woman presented with chest discomfort and New York Heart Association class 2 dyspnea when she climbed stairs 2 weeks ago. She was not taking any medication except hypnotics, and her only cardiovascular risk factor was old age. Although she had had 3 natural childbirths, she had no history of surgery or blood transfusions. No specific findings were observed upon physical examination, electrocardiography (ECG), or chest x-ray imaging, and cardiac biomarkers were within the normal range, but the D-dimer, prothrombin time (PT), partial thromboplastin time (PTT), and activated PTT levels were prolonged. Transthoracic echocardiography showed a normal left ventricular ejection fraction and no regional wall motion abnormality. On the basis of the exercise-induced ECG changes in the treadmill exercise test, coronary angiography was planned to conduct decision-making for appropriate management and prognosis assessment (class I, level of evidence B).^[5]^ After 300 mg of aspirin and 180 mg of ticagrelor were administered, coronary angiography was performed via the right radial artery. A significant stenosis was noted in the left anterior descending coronary artery and right coronary artery; thus coronary stenting was performed successfully using DES stents (Fig. 1). Unexpectedly, D-dimer, PT, and PTT prolongation were maintained at 6 and 24 hours after coronary stenting, and hemoglobin (HgB) decreased from 11.3 to 9.5 g/dL. Although ecchymosis and oozing were present at the right radial artery puncture site, no evidence of bleeding was observed. Aspirin (100 mg daily) and ticagrelor (90 mg twice daily) were administered to prevent a stent thrombosis. The test values to identify the causes of prolonged coagulopathy fell within the normal range. Epistaxis and blood-tinged sputum occurred on day 3 after coronary stenting. Because HgB had dropped to 8.5 g/dL, chest and abdominal computed tomography scans were performed to confirm the possibility of internal bleeding; however, no abnormal findings were observed except aortic calcification. The antiplatelet therapy measured using the Multiplate Analyzer (Roche Diagnostics, Mannheim, Germany) was adequate, but the daily dose of the antithrombotic agent was reduced to 100 mg of aspirin and 90 mg of ticagrelor. After transfusing 2 units of packed red cells, HgB remained >10 g/dL (Fig. 2). On day 5, factor V activity was 14% (reference range 60%–120%), but other laboratory findings, including factor VIII activity, were all within normal ranges; she was diagnosed with F5D based on low factor V activity. Hematochezia occurred on day 6, and ischemic colitis was confirmed via sigmoidoscopy (Fig. 3). After fasting, except antithrombotic agents and intravenous antibiotic treatment, a general diet was made available starting on day 8, and HgB was maintained consistently above 10 g/dL. However, PT, PTT, and D-dimer prolongation persisted. The area under the curve (AUC) was 20 (reference range 1–41) for ticagrelor and 18 (reference range 1–29) for aspirin with the daily maintenance dose of 90 mg of ticagrelor and 100 mg of aspirin, with which she was discharged on day 10.

The patient was readmitted due to recurrent epistaxis, hemoptysis, and cough on day 26 after coronary stenting. HgB and the AUCs of aspirin and ticagrelor were 10.7 g/dL, 19, and 22, respectively, whereas PT, PTT, and factor V activity showed no significant changes (Fig. 2). Due to the recurrent epistaxis and oozing of the nasal mucus membrane, the aspirin was stopped and only the daily maintenance dose of 90 mg of ticagrelor was maintained. HgB remained constant at 9.5 g/dL, and epistaxis and hemoptysis both stopped. The factor V activity and AUC value of ticagrelor 5 days after stopping aspirin were 12 and 52, respectively. After symptoms improved, she was discharged on day 35 with only 90 mg of ticagrelor daily.

The HgB level was 10 g/dL and the AUC of ticagrelor was 24 at 9 weeks (Fig. 2), and the patient led healthy life for 9 months without any recurrent symptoms. At the 9-month follow-up, the AUC of ticagrelor was stabilized at 18 and the other test results were more stabilized compared with those at the start of coronary stenting (Fig. 2).

## 3 Discussion

We present the case of a patient with F5D and unstable angina who underwent coronary stenting and antithrombotic maintenance therapy with a single antiplatelet agent due to recurrent multiple mucosal bleeding shortly after coronary stenting. In particular, for the first time, we have described maintenance therapy after coronary stenting in an F5D patient. As F5D is a rare hematological disease with an incidence of approximately 1 in a million, it has no established treatment guidelines. Furthermore, there are no guidelines or studies on antithrombotic therapy after coronary stenting in F5D patients.

Although F5D is defined as a mild and severe disease in cases with activity >5% and <1%, respectively, factor V activity does not necessarily predict bleeding severity or clinical features, and many patients may present with a mild disease even with factor V activity of <1%.^[2]^ However, F5D therapy depends on the factor V activity level, and is classified as bleeding control and the removal of inhibitors or antibodies of factor V. FFP or platelet transfusion is recommended to control bleeding. In particular, transfusing FFP is recommended to maintain factor V activity at >25% to 30% in cases of bleeding, invasive testing, or surgery.^[3,6]^ In this case, FFP was not transfused because coronary stenting was performed without knowledge of the degree of factor V activity or the existence of F5D. We recognized F5D due to hemoptysis, epistaxis, and hematochezia after coronary stenting. However, we first planned to reduce or modify the dosage of antithrombotic drugs due to the risk of stent thrombosis. This strategy was based on previous reports in which factor V activity was not related to bleeding severity.^[2,6]^ Moreover, successful childbirth delivery was possible without an FFP transfusion, and the success rate for bleeding control was low with FFP transfusion.^[3,6]^ Fortunately, mucosal bleeding in the present case was controlled by adjusting the antithrombotic drug dose. Treatments for the inhibitors or antibodies to factor V are another option and include plasmapheresis, immunosuppressants, steroids, intravenous globulins, and a monoclonal antibody.^[2]^ Such therapies were not used in our case because the inhibitors and autoantibodies for factor V were unknown and such therapies have weak evidence.

Another notable finding is the effect of 100 mg of aspirin daily on mucosal bleeding in F5D patients. P2Y_12_ generally plays a key role in dual antiplatelet therapy (DAPT). In this instance, the AUC values of aspirin and ticagrelor were 8 and 10, with 100 mg of aspirin daily and 90 mg of ticagrelor twice a day, respectively. The AUC value for ticagrelor was 22 for a daily regimen of 100 mg of aspirin and 90 mg of ticagrelor, but the AUC for ticagrelor increased to 52 when only the 90 mg of ticagrelor was maintained. This observation emphasizes that ticagrelor's antithrombotic effect is affected by aspirin. In fact, the Platelet Inhibition and Patient Outcomes trial showed that daily combination of 100 mg of aspirin and ticagrelor was superior to aspirin and clopidogrel combined, but the use of 300 mg/day of aspirin wiped out the benefit.^[7]^ Moreover, Kirkby et al^[8]^ demonstrated that aspirin provides additional antiaggregatory effects when only a partial P2Y_12_ blockade is achieved. Daily 90 mg of ticagrelor administration means incomplete P2Y_12_ inhibition and 100 mg of aspirin has the enhancing effect of suppressing platelet aggregation for the daily dose of 90 mg of ticagrelor. Aspirin discontinuance with a daily dose of 90 mg of ticagrelor causes ticagrelor to have a weakened antiplatelet effect, which can be expressed as an increased AUC value for ticagrelor. Meanwhile, it is unclear whether the disappearance of mucosal bleeding after stopping aspirin can be ascribed to the antithrombotic effect of aspirin itself or its excessive dose (100 mg/day). However, as the daily dose of 100 mg of aspirin may have had a strong antithrombotic effect in a patient with F5D and mucosal bleeding; aspirin may have to be stopped to control mucosal bleeding when simultaneously taking a P2Y_12_ receptor blocker.

The initial loading dose of aspirin, a P2Y_12_ receptor blocker, and intravenous heparin were used to prevent thrombosis (particularly stent thrombosis) during coronary stenting and standard maintenance antithrombotic therapy after implanting a DES composed of DAPT (aspirin and a P2Y_12_ receptor blocker). Generally, the optimal range for the antithrombotic effect is known as mid-third platelet inhibition.^[4,9]^ F5D was detected after the coronary stenting procedure; thus, the routine DAPT protocol was applied to coronary intervention, and a decrease in HgB was observed that started on the day of coronary stenting. This finding indicates that a weaker antithrombotic effect is recommended for coronary intervention in patients with F5D. In particular, recurrent mucosal bleeding for daily doses of 90 mg of ticagrelor and 100 mg of aspirin suggests that a weakened antithrombotic effect is also required during maintenance therapy after coronary stenting. The disappearance of mucosal bleeding after stopping the 100 mg of aspirin means that ticagrelor may be more effective than aspirin for controlling mucosal bleeding and maintaining antithrombosis in F5D patients after coronary stenting.

Another unusual feature of this case beyond the antithrombotic therapy is ischemic colitis. Unlike mucosal bleeding, such as epistaxis or hemoptysis, ischemic colitis is ascribed to tissue hypoperfusion through the reduced blood supply to the mesenteric artery rather than bleeding tendency or a coagulation disorder. In this patient, tissue hypoperfusion may occur due to transient blood flow reduction, suggesting old age, abdominal aortic calcification, and a sudden drop in the hemoglobin level. Thus, it is unclear whether the ischemic colitis seen here was related to F5D. Furthermore, additional evidence is needed regarding whether antithrombotic therapy presents a risk factor for F5D patients developing ischemic colitis.

Finally, the result of mixing tests in which test plasma is combined with normal plasma was normal, and the absence of inhibitors, including anticoagulants, lupus anticoagulant, or another inhibitor type, was documented. However, we neither performed genetic testing nor investigated autoantibodies for factor V due to the testing cost and the fact that it would provide no additional benefit.

## 4 Conclusions

We report a patient with F5D and multiple mucosal bleeding events after coronary stenting who was successfully treated by reducing the daily antithrombotic maintenance dose from DAPT to 90 mg of ticagrelor.
